# Advantages and Limitations of Gene Therapy and Gene Editing for Friedreich’s Ataxia

**DOI:** 10.3389/fgeed.2022.903139

**Published:** 2022-05-17

**Authors:** Anusha Sivakumar, Stephanie Cherqui

**Affiliations:** Division of Genetics, Department of Pediatrics, University of California, San Diego, San Diego, CA, United States

**Keywords:** Friedreich’s ataxia, CRISPR/Cas9 gene editing, AAV, gene therapy, hematopoietic stem and progenitor cells, gene editing

## Abstract

Friedreich’s ataxia (FRDA) is an inherited, multisystemic disorder predominantly caused by GAA hyper expansion in intron 1 of frataxin (*FXN*) gene. This expansion mutation transcriptionally represses *FXN*, a mitochondrial protein that is required for iron metabolism and mitochondrial homeostasis, leading to neurodegerative and cardiac dysfunction. Current therapeutic options for FRDA are focused on improving mitochondrial function and increasing frataxin expression through pharmacological interventions but are not effective in delaying or preventing the neurodegeneration in clinical trials. Recent research on *in vivo* and *ex vivo* gene therapy methods in FRDA animal and cell models showcase its promise as a one-time therapy for FRDA. In this review, we provide an overview on the current and emerging prospects of gene therapy for FRDA, with specific focus on advantages of CRISPR/Cas9-mediated gene editing of *FXN* as a viable option to restore endogenous frataxin expression. We also assess the potential of *ex vivo* gene editing in hematopoietic stem and progenitor cells as a potential autologous transplantation therapeutic option and discuss its advantages in tackling FRDA-specific safety aspects for clinical translation.

## Introduction

Friedreich’s ataxia (FRDA) is the most common inherited human ataxia with an incidence of 1 in 50,000 individuals. FRDA is an autosomal recessive disorder resulting from deficiency of the mitochondrial protein, frataxin (FXN) (Campuzano et al., 1996; Delatycki et al., 2000). The most common mutation is the expansion of GAA trinucleotide repeats in the first intron of the gene leading to decreased *FXN* transcription due to heterochromatin formation and/or epigenetic modification (Gottesfeld, 2019). The protein level in patients ranges between 5 and 35% of the levels in healthy individuals (Gottesfeld et al., 2013). The age of onset and severity of the symptoms correlate with the number of repeats, which can vary between 66 and 1,700 (Dürr et al., 1996; Epplen et al., 1997). Point mutations have also been described in rare cases, but always at the heterozygote state along with a GAA expansion mutation (Campuzano et al., 1996; Li et al., 2013). Although the exact function of FXN is still unclear, it is predicted to assist in the biogenesis of mitochondrial iron-sulfur clusters (Tsai and Barondeau, 2010; González-Cabo and Palau, 2013). Thus, frataxin deficiency results in altered cellular iron metabolism, increased mitochondrial iron load, decreased mitochondrial energy production and biogenesis as well as increased oxidative stress.

FRDA is a progressively lethal multi-systemic disease. The primary pathological cause of the neuropathy is the progressive loss of large sensory neurons in the dorsal root ganglia (DRG) affecting the peripheral and central nervous systems (CNS) (Pandolfo, 2009; Koeppen and Mazurkiewicz, 2013). This progressive neurodegeneration leads to loss of motor skills and muscle degeneration, and ultimately the inability to walk, within 10–15 years of onset. Heart abnormalities cause premature death in 60–80% of the affected individuals and is usually the primary cause of death (Weidemann et al., 2013; Perdomini et al., 2014).

Currently, there are no effective treatment for FRDA. Clinical trials with antioxidants (idebenone and coenzyme Q_10_), iron chelators (deferipone) and epigenetic modulators (RG2833, nicotinamide) failed to prove efficacy in patients (Arpa et al., 2014; Libri et al., 2014; Soragni et al., 2014) but omaveloxolone, an *NRF2* activator, improved neurological functions in Phase 2 clinical trial (Lynch et al., 2021). Gene therapy for FRDA is also under active investigation and studies of additive gene therapy using viral vectors carrying *FXN* cDNA have reported promising outcomes *in vitro* and *in vivo.* An adeno-associated virus (AAV)-based gene therapy product, LX2006, targeting FRDA cardiomyopathy, was recently approved for Phase 1/2 clinical trial by the United States Food & Drug Administration (FDA) following promising preclinical studies (Salami et al., 2020). An alternative approach for gene therapy is gene editing, which allows manipulating eukaryotic genomes using target-specific engineered nucleases. Gene editing has the key advantage of correcting the defective gene *in situ,* keeping their internal regulation system intact. Several gene editing nucleases like the zinc finger nucleases (ZFN), transcription activator-like effector (TALE) nucleases (TALENS), and CRISPR/Cas9 (clustered regularly interspaced short palindromic repeats-associated nuclease Cas9) exist and are continuously evolving with new generation variants (Gaj et al., 2013). CRISPR/Cas9 is gaining more traction in recent years due to its specificity, efficiency in editing, and simplicity in design.

Post-mitotic cells like neurons and cardiomyocytes, the primary cell types affected by FRDA, pose significant challenges for gene therapy methods and in multisystemic disorders, systemic expression of the protein is critical for rescue of disease phenotype. In such cases, *ex vivo* gene therapy using hematopoietic stem and progenitor cells (HSPC) are versatile, safe and efficient delivery vehicles, and have been widely used in regenerative and cell replacement therapy including neurodegenerative disorders (Porter et al., 2011; DiGiusto et al., 2013; Drakopoulou et al., 2013; Zhang et al., 2013; Eichler et al., 2017a; Massaro et al., 2021). These cells can migrate to and differentiate into tissue-specific macrophages for delivering organelles such as mitochondria and lysosomes, proteins, ions and microRNAs (Naphade et al., 2015; Dupont et al., 2018). Thus, a single infusion of gene-corrected stem cells residing in the bone marrow niche will become a reservoir of healthy cells for lifespan of the patient. This is a potentially viable approach for FRDA for ensuring sustained systemic delivery of frataxin to the injured organs. In this review, we report on the studies and progress being made in *in vivo* gene therapy, gene editing and *ex vivo* gene therapy for FRDA with a special focus on the potential of *ex vivo* gene editing as a new therapeutic avenue for FRDA, and discuss its advantages in tackling FRDA-specific safety aspects for clinical translation.

### Additive Gene Therapy for Friedreich’s Ataxia

Because FRDA is a monogenic disease caused by reduction of *FXN* expression, gene addition using viral vectors represent a promising strategy. Lentiviruses (LV), herpes simplex virus type 1 (HSV-1) and AAVs have been used as delivery vehicles of *FXN* for *in vivo* and *in vitro* studies of FRDA. LV expressing human *FXN* (h*FXN*) under CMV (human immediate-early cytomegalovirus) promoter and HSV-1 carrying full genomic DNA of *FXN,* with its endogenous promoter, enhancer elements and introns, have shown therapeutic benefits in human fibroblasts derived from FRDA patients, resulting in the partial or complete restoration of the normal cellular phenotype in response to oxidative stress (Fleming et al., 2005; Gomez-Sebastian et al., 2007). Similarly, HSV-1 vector carrying h*FXN* cDNA, injected into the brainstem of a conditional neuronal *Fxn*-knockout mice, having 20–60% reduced *FXN* expression in olivary neurons, led to the recovery of the motor coordination of the treated mice by restoring *FXN* expression to that of physiological levels (Lim et al., 2007). The promise of AAV9 serotype carrying human *SMN1* in improving survival and motor functions of wheelchair-bound children with spinal muscular atrophy (SMA) (Mendell et al., 2017) spurred translation of several explorative research to clinical trials for neurodegenerative diseases with AAVs. These are single-stranded DNA viruses suitable for CNS associated diseases as they can infect both dividing and non-dividing cells with selective serotypes like the AAV9 and AAVrh10 capable of crossing the blood brain barrier (BBB), the major hurdle in gene therapy for neurodegenerative diseases (Kantor et al., 2014; Zhang et al., 2011). Both AAV9 and AAVrh10 have seen applications for FRDA, either carrying the *FXN* transgene or transcription activators that induce endogenous *FXN* expression (Table 1). Trembley and colleagues used AAV9 containing TALE gene fused with a transcription activator domain (TAD) to target the proximal promoter of *FXN* gene and induced its expression *in vitro* and *in vivo*. A series of studies with TALEs and TADs led to the identification of TALE-VP64, a tetrameric repeat of VP16 protein of Herpes simplex virus that acts as a strong transcription activator upon binding to the promoter sequence. Under the control of CAG promoter, TALE-VP64 enhanced *FXN* gene expression by 1.6–1.9-fold and mature frataxin protein expression by 1.4-fold in FRDA patient-derived fibroblasts (Tremblay et al., 2012). The same TALE-VP64, when delivered intraperitonially in the YG8R mouse model of FRDA using AAV9 vector, increased h*FXN* mRNA and protein expression levels in the liver, heart, and skeletal muscle (Chapdelaine et al., 2016). YG8R mice are the original transgenic humanized animal model of FRDA generated by Dr. Pook that expresses two human *FXN* transgenes, containing 82 and 190 GAA repeats, in a murine frataxin null background (fxn^−/−^FXN^+^) (Al-Mahdawi et al., 2006). However, despite injection of high viral copy numbers, AAV9-carrying TALE-VP64 expression in the brain was low and did not improve frataxin expression. The therapeutic effect of AAV9 vector containing h*FXN* transgene was also tested in the MCK-Cre and NSE-Cre mouse models of FRDA. MCK-Cre mice is a cardiac and striated muscle conditional *Fxn*-knockout (KO) mouse model with *Cre* transgene being expressed under the *M*uscle *C*reatine *K*inase promoter, and the NSE-Cre is conditional *Fxn*-KO in neurons using the *N*euron-*S*pecific *E*nolase promoter, with partial KO in other organs (Puccio et al., 2001). Both these models are considered acute models of FRDA because they display early and severe onset of symptoms with a short-life expectancy of < 40 days caused by complete absence of *Fxn* in the targeted tissues. Intraperitoneal injection of AAV9 vector containing h*FXN* transgene expressed under CB (CMV enhancer/chicken beta-actin) promoter increased the life span of NSE-Cre mice by 3-fold and MCK-Cre by 2.7 fold, improved locomotion in NSE-Cre mice, and reduced cardiac hypertrophy and improved the overall cardiac function in MCK-Cre mice (Gérard et al., 2014). AAVrh10, a serotype of AAV isolated from rhesus monkey, is shown to have similar tropism to the CNS than AAV9 (Tanguy et al., 2015). When AAVrh10 containing h*FXN* expressed under CAG promoter was intraperitonially injected into MCK-Cre mice, it increased frataxin expression in the heart, leading to preservation of the hemodynamic parameters and cardiac output, and also complete reversal of the cardiomyopathy after disease onset (Perdomini et al., 2014). Indeed, cardiomyocytes with severe energy failure and ultrastructure disorganization could be rescued and remodeled by this gene therapy approach. Similarly, Salami et al. (2020) also demonstrate the therapeutic potential of AAVrh10-h*FXN* for the cardiac phenotype including improved cardiac ejection fraction and myocardial fractional shortening using a cardiac-specific partial *Fxn* knockout model, the αMyhc mice. These mice have 51% reduced expression of frataxin in the heart and associated cardiac pathology, as the Cre expression is driven by a cardiac specific αMyhc promoter.

**TABLE 1:** Feasibility of gene therapy methods in increasing FXN expression, *in vitro and in vivo*.

Gene addition (viral vectors]							
Viral vector	FRDA model	Viral genome (vg)	*FXN* mRNA fold change				References
			Brain	Heart	Muscle	Liver	
AAV9—TALE_VP64_	YG8R mice	1.2 × 10^11^	No change			Yes	Chapdelaine et al. (2016)
		6 × 10^12^	No change	Yes	Yes	Yes	
AAV9-hFXN	MCK-Cre and NSE-Cre	6 × 10^11^	—				Gérard et al. (2014)
AAVrh10-FXN	αMyhc	1 × 10^11^	—				Salami et al. (2020)
AAVrh10–CAG–hFXN	MCK-Cre	5.4 × 10^13^ vg/kg	Not detected	High	No change	High	Perdomini et al. (2014)
HSV1-hFXN	loxP [frda] neuronal	1.4 × 10^4^ infectious vector units	—				Lim et al. (2007)
	conditional KO						
**GENE EDITING (Nucleases)**							
**Nucleases**	**FRDA Model**	**Delivery method**	**Editing efficiency**	***FXN mRNA*** **fold change**	**References**		
Zinc-finger nucleases	FRDA patient fibroblasts and lymphoblasts	Transfection of *in vitro* transcribed mRNA	Yes	Yes	Li et al. (2015); Li et al. (2019)		
	FRDA fibroblasts→iPSCs→Neurons			Yes			
	FRDA fibroblasts→iPSCs→Cardiomyocytes			Yes			
Cas9 nuclease	YG8R and YG8sR fibroblasts	Lipofectamine mediated transfection of pxPuro plasmid carrying Cas9 and gRNA	Yes	Variable with the gRNA used	Ouellet et al. (2017)		
Cas9 nuclease	FRDA fibroblast→iPSC→DRG organoids	Lipofectamine mediated co-transfection of pCAG-Cas9-Puro for Cas9 and LV-U6-sgRNA-EF1α-Blast for gRNA	—	Normalized to the levels in healthy control cells	Mazzara et al. (2020)		
Cas9 nuclease	FRDA Lymphoblasts	Electroporation of RNP complex of Cas9, two gRNAs and electroporation enhancer	Yes	Normalized to the levels of carriers or healthy control cells	Rocca et al. (2020)		
	CD34^+^ HSPCs from FRDA patients		Yes				

These preclinical studies demonstrate the potential of viral vectors as delivery vehicles for frataxin transgene, leading to disease phenotype rescue, either cardiac or neurological. However, both the clinical complications must be treated in FRDA as the patients lose their motor function with progressively worsening cardiac functions. With gene additive therapy, it is also critical to ensure that frataxin expression is tightly controlled. Indeed, frataxin is expressed at a relatively low level even in healthy individuals (Campuzano et al., 1996; Campuzano et al., 1997), and while some studies showed that overexpression of frataxin was not harmful and even had positive effects (Ristow et al., 2000; Shoichet et al., 2002; Miranda et al., 2004; Schulz et al., 2010), others showed that this was deleterious (Navarro et al., 2011; Wang et al., 2014). Transgenic flies overexpressing human frataxin (h*FXN*) had reduced viability with neurologic and muscular defects (Navarro et al., 2011), and overexpression of frataxin homologue in a yeast model enhanced oxidative stress and iron accumulation (Wang et al., 2014). Further, AAVrh10-mediated frataxin overexpression in the MCK-Cre mice heart promoted mitochondrial ultrastructural damages, impaired respiratory complex functions, and caused myocardial fibrosis (Belbellaa et al., 2020). The study also showed overexpression of frataxin even with lower dose of AAVrh10-h*FXN* still led to cardiotoxicity. More recently, a Phase 1 clinical study on recombinant fusion protein delivering functional frataxin to mitochondria (CTI 1601) was put on hold as the study investigators reported mortality of non-human primates in toxicological studies in the high dose cohort (Vyas et al., 2012; LarimarTherapeutics, 2022).

In addition to the toxicity associated with frataxin overexpression, *in vivo* gene therapy using viral vectors inherently poses potential safety and logistic concerns: 1) localized delivery by direct viral injection to affected sites poses challenges in accessing sites such as heart, brain, and DRG, and leads only to tissue-specific rescue; 2) systemic AAV delivery remains difficult in humans due to the high levels of vector necessary, leading to vector synthesis and safety concerns including potential immune reaction. This is particularly concerning in light of the recent reports on severe adverse events and deaths in AAV-based gene therapy clinical trials. Three participants in the high dose cohort of the X-linked Myotubular Myopathy clinical trial developed liver dysfunction and sepsis, leading to their death (Morales et al., 2020; AstellasPharma, 2021). More recently, an additional participant belonging to the low dose cohort developed hepatic abnormalities and was reported dead and investigation into the cause is underway. The Phase 1b clinical trial for Duchenne muscular dystrophy clinical trial is on hold due to the death of a subject (Bryson, 2021), and severe adverse events such as low platelet counts and kidney injury were also reported on another trial (SolidBiosciences, 2019). Similarly, in the SMA clinical trial, thrombotic microangiopathy were seen in 3 infants that may be due to an immune reaction to the therapy (Chand et al., 2021). Therefore, while gene addition mediated by viral vectors holds promising therapeutic potential, it is essential to address the toxicity associated with sustained systemic frataxin expression and high dose AAV vector for future clinical application of this strategy.

### Gene Editing for Friedreich’s Ataxia

**FIGURE 1:**
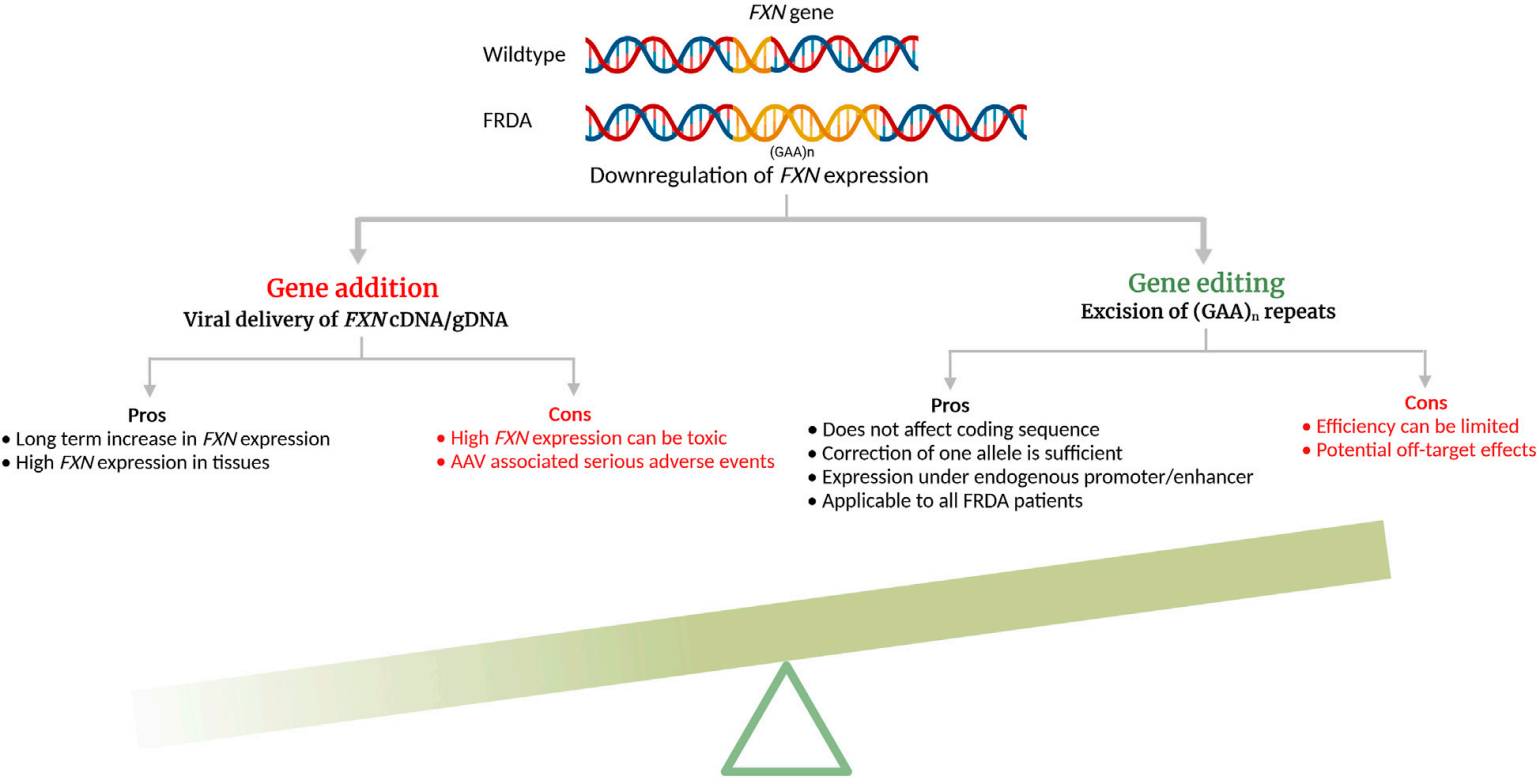
Pros and cons of additive gene therapy and gene editing for Friedreich’s ataxia (cDNA—complimentary DNA; gDNA—genomic DNA). The schematic was created with BioRender.com.

Genome editing has been in the development for several decades but the advent of CRISPR/Cas9 and its ease-of-use have rendered preclinical studies and clinical translation for multisystemic diseases much more accessible. All three engineered nucleases, ZFN, TALEN, and CRISPR/Cas9 induce double strand DNA breaks (DSBs) in targeted DNA that are then repaired by the cell’s innate DNA repair mechanisms, the homology-directed repair (HDR) or non-homologous end joining (NHEJ) (Li et al., 2020). While NHEJ is error-prone and occur during any cell cycle phase, HDR is more efficient in repairing and preferentially occurs during S or G2 phase, using sister chromatid as the template. In FRDA, because the GAA expansion mutation is located in the intron 1 of the *FXN* gene, removal of the repeats can be done by creating DSBs in the 5′- and 3′- sites flanking the repeat region without disturbing the coding sequence and be maintained under its endogenous promoter/enhancers. In addition, mutation carriers do not display the disease phenotype, thus correcting one allele should lead to cellular phenotype correction (Coppola et al., 2011). Genome editing approaches for reactivating endogenous *FXN* have been studied for FRDA, both *in vitro* and *in vivo* (Table 1) and its significant advantages are schematized in Figure 1.

Nucleofection of *ZFN* mRNA flanking the GAA repeats mutation in the intron 1 of *FXN* led to an editing efficiency of 2.3% and 6.7% in single clones derived from FRDA lymphoblasts and fibroblasts, respectively, and increased its mRNA expression by ∼2.5–4.5 fold in both the lines (Li et al., 2015). When the edited fibroblasts were reprogrammed into induced pluripotent stem cell (iPSC) -derived neurons, they retained allele correction, and exhibited improved mitochondrial and cellular functions (Li et al., 2015). Similarly, when differentiated into iPSC-derived cardiomyocytes, *FXN* mRNA expression was increased ∼3-fold and genes associated with cardiac hypertrophy development were alleviated (Li et al., 2019). While clinical trials with ZFNs are still undertaken for mucopolisaccharidosis II, HIV/AIDS, transfusion dependent β-thalassemia and others (Tebas et al., 2014; First *in vivo* human genome, 2018; Walters et al., 2021), the design complexity and gene editing efficacy level have limited their widespread application.

The CRISPR/Cas9 system has supplanted ZFNs in terms of higher efficiency, minimal off-target effects, and a simplified three-component system, offering a modular way of editing the genome. CRISPR/Cas9 allows robust RNA-guided genome modifications in multiple eukaryotic systems (Cho et al., 2013; Cong et al., 2013; Mali et al., 2013) using a target specific CRISPR RNA (crRNA) and a universal trans-activating CRISPR RNA (tracrRNA) that form the guideRNA (gRNA) for specific cleavage by Cas9 (Karvelis et al., 2013). The tracrRNA and crRNA can be chemically fused to form a single guide RNA (sgRNA) that reduces the CRISPR/Cas9 technology to a two-component system. Delivery of AAV vector carrying both Cas9, under CMV promoter, and gRNA, under U6 Pol III promoter, in murine fibroblasts isolated from YG8R and YG8sR mice, led to editing efficiency ranging from 21.6 to 50% depending on the gRNA (Ouellet et al., 2017). YG8sR mice are derived from YG8R and carry a single h*FXN* transgene with 190 GAA repeats (Anjomani Virmouni et al., 2015). Similarly, LV mediated delivery of Cas9, under CAG promoter, and gRNA, under U6 promoter, were used by Mazzara *et al.* to show that excision of complete intron 1 as opposed to the GAA expansion only, overcame the epigenetic repression and improved *in vitro* survival of DRG organoids (DRGO) differentiated from FRDA patient-derived iPSC clones (Mazzara et al., 2020). These DRGOs also demonstrated improved mitochondrial biogenesis and function with enhanced axonal spreading. Taken together, these *in vitro* studies showed that gene editing to remove the GAA hyper expansion is an efficient strategy for FRDA leading to increased *FXN* mRNA and protein expression, and cellular phenotype improvement, and CRISPR/Cas9 is particularly attractive for this purpose. However, despite its higher efficiency, its use *in vivo* is still limited due to potential safety concern associated with vector-mediated delivery of Cas9 that would result in long-term expression of this protein, increasing the risks for off-target activity (Pattanayak et al., 2013; Merkle et al., 2015). Indeed, off-target effects are highly reliant on the specificity of the gRNAs to the target sequence and the duration of the editing system within the cells and are extensively reviewed elsewhere (Listgarten et al., 2018; Atkins et al., 2021; Vicente et al., 2021). In contrast, the use of *ex vivo* gene editing would have the critical benefits of having the Cas9 delivered transiently in cells that can be characterized for gene editing efficiency, potential off-target effects and other safety features, prior to transplant.

### *Ex Vivo* Gene Editing for Friedreich’s Ataxia

*Ex vivo* gene therapy using hematopoietic stem and progenitor cell (HSPC) has been on the rise for treating inherited, immunological, metabolic, and neurodegenerative disorders (Massaro et al., 2021; DiGiusto et al., 2013; Drakopoulou et al., 2013; Eichler et al., 2017a; Porter et al., 2011; Zhang et al., 2013; Tucci et al., 2021). *Ex vivo* HSPC gene therapy has potential key advantages: 1) it avoids immune reaction during an autologous transplantation procedure (Drysdale et al., 2020), 2) it may treat all the complications by a single infusion of hematopoietic stem cells (Epah and Schäfer, 2021); 3) *ex vivo* gene modification of the patients’ cells will occur in a controlled environment allowing cell characterization prior to transplantation (Soni and Kohn, 2019); 4) it potentially provides once-in-a-lifetime intervention as engrafted, gene modified HSPCs will constitute a long-term reservoir of repopulating healthy cells in the bone marrow; stable transgene expression of over 15 years is reported for severe combined immunodeficiency caused by adenosine deaminase deficiency (ADA-SCID) (Cicalese et al., 2016); 5) HSPC-derived monocyte/macrophages can cross the BBB and engraft long-term in the CNS as microglia-like cells in the context of neurodegenerative disorders and after myeloablative conditioning (Capotondo et al., 2012; Peterson et al., 2019). and, 6) HSPC-derived macrophages and microglia can deliver lacking protein/enzyme to the disease cells in the injured tissues (Tan et al., 2019). Clinical trials using gene-modified autologous CD34^+^ HSPCs are being undertaken for genetic diseases such as X-SCID, ADA-SCID, Wiskott-Aldrich syndrome, metachromatic leukodystrophy, X-linked cerebral adrenoleukodystrophy, and mucopolysaccharidosis type I (Gentner et al., 2021; Mamcarz et al., 2019; De Ravin et al., 2016; Kohn et al., 2021; Magnani et al., 2022; Ma et al., 2021; Ferrua and Aiuti, 2017; Morris et al., 2017; Biffi et al., 2013; Eichler et al., 2017b; Fumagalli et al., 2022). Currently, our lab is conducting a phase 1/2 clinical trial for cystinosis (ClinicalTrials.gov Identifier: NCT03897361), a multisystemic lysosomal storage disorder, characterized by accumulation of cystine in all tissues and due to mutations or deletions in *CTNS* gene, encoding a lysosomal cystine transporter (Cherqui, 2021). Single infusion of *ex vivo* gene-corrected HSPCs using a self-inactivated lentiviral vector carrying *CTNS* cDNA in the mouse model of cystinosis led to long-term preservation of the kidney (Yeagy et al., 2011), eye (Rocca et al., 2015) and thyroid (Gaide Chevronnay et al., 2016) functions. The mechanism underlying this therapeutic effect involves the tissue engraftment of HSPCs and differentiation into macrophages, which provide “healthy lysosomes” carrying the functional cystinosin to the diseased cells *via* extension of tunneling nanotubes (TNTs) (Naphade et al., 2015). Because mitochondria can also be transferred through TNTs (Domhan et al., 2011; Vallabhaneni et al., 2012), we tested the impact of HSPC transplantation on FRDA using the YG8R murine model (Rocca et al., 2017). A single systemic transplantation of wildtype bone marrow HSPCs in YG8R mice prevented the neurological complications and muscle weakness in the treated mice, with functional, histological and biochemical properties comparable to WT mice as opposed to non-treated YG8R mice or treated with YG8R HSPCs. Cardiac iron deposits were also prevented in old YG8R mice. Abundant HSPCs engrafted into affected tissues and differentiated into microglia in brain and spinal cord, and macrophages in DRG, heart and muscle, and led to frataxin transfer to the diseased neurons and myocytes. Another study (Kemp et al., 2018) subsequently reported similar results where transplantation of wild type bone marrow (BM) cells to YG8R FRDA mice improved motor coordination, rescued neurobehavioral deficits and resulted in engraftment of bone marrow-derived macrophages/microglia in DRG, spinal cord and cerebellum. Altogether, these data represent the proof of concept that the different complications associated with FRDA could be treated by a HSPC transplantation. However, gene addition in HSPCs would potentially lead to the same toxicity issue associated with FXN overexpression and thus, gene editing represents a better option.

Autologous transplantation of CRISPR/Cas9–mediated gene edited CD34^+^ HPSCs has seen its first success in the rare diseases, β-thalassemia and sickle cell disease (SCD) (Frangoul et al., 2021a). For both diseases, targeted disruption in the specific transcription factor binding site on *BCL11A* erythroid enhancer leading to downregulation of BCL11A in CD34^+^ HSPCs, reactivated the production of fetal hemoglobin in adult-stage erythroid cells. These cells showed gene editing ranging from 9.5 to 87.0% (Wu et al., 2019). After a year, the patients had high level of fetal globin, and were transfusion-free for β-thalassemia, and with no episode of vaso-occlusion for SCD. A similar approach was undertaken by our group to remove the GAA repeats in FRDA patients’ CD34^+^ HSPCs using RNP mediated delivery of Cas9 protein pre-complexed with gRNA (Rocca et al., 2020). RNP complexes are well tolerated by CD34^+^ HSPCs [Table 2, (Cromer et al., 2018)] and addresses the clinical concerns with cloning *Cas9* and gRNAs on vectors and associated viral genotoxicity. In addition, it was previously shown that transfected Cas9/RNP complex cleave chromosomal DNA almost immediately after delivery and are degraded rapidly in cells, reducing off-target effects, and increases gene editing efficiency in mouse and human HSPCs: ∼60% and ∼75%, respectively, with good cell viability: ∼80% and ∼69%, respectively (Gundry et al., 2016; Lattanzi et al., 2019). RNP complexes are also widely used in preclinical studies of CD34^+^ HSPC mediated therapy, some of which are listed in Table 2.

**TABLE 2:** Ribonucleoprotein (RNP) mediated delivery of CRISPR/Cas9 in patient derived CD34^+^ HSPCs.

Disease	CRISPR/Cas9 delivery	Efficiency	References
X-Linked Hyper-IgM Syndrome	RNP	∼33%	Kuo et al. (2018)
X-linked chronic granulomatous disease	RNP	>21%	De Ravin et al. (2017)
Fanconi Anemia	RNP	23.33%	Román-Rodríguez et al. (2019)
Wiskott - Aldrich Syndrome	RNP	60%	Rai et al. (2020)
Sickle cell disease	RNP	24.5 ± 7.6%	Park et al. (2019)
Sickle cell disease	RNP	32%	DeWitt et al. (2016)
Severe Congenital Neutropenia	RNP	40–56%	Tran et al. (2020)
β-thalassemia & Sickle cell disease CRISPR/Cas9 clinical trial	RNP	∼80%	Frangoul et al. (2021b)
Mucopolysaccharidosis Type I	RNP	76 ± 8%	Gomez-Ospina et al. (2019)

**FIGURE 2:**
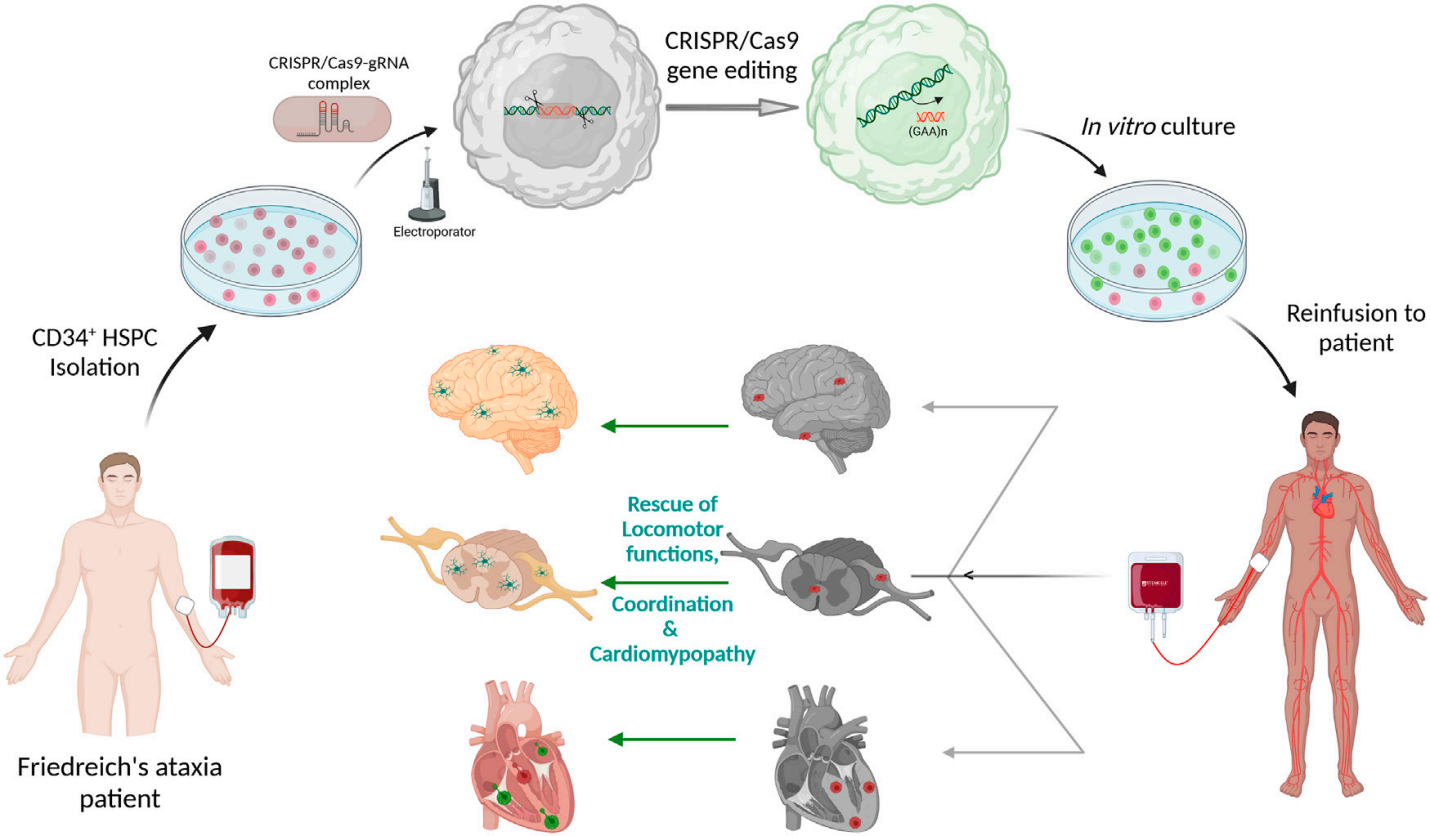
Schematic representation of Autologous transplantation of CRISPR/Cas9 gene-edited CD34^+^ Hematopoietic Stem and Progenitor Cells (HSPCs) for Friedreich’s ataxia. CD34^+^ HSPCs isolated from peripheral blood of Friedreich’s ataxia patient will be electroporated with pre-complexed CRISPR/Cas9-guideRNA ribonucleoprotein complex for excision of the (GAA)n repeat sequences in intron 1 of *FXN*. These gene-edited HSPCs will be put back in culture and then be reinfused to the same patient. The gene-corrected HSPCs are expected to engraft into the disease tissues such as the heart, brain, spinal cord and dorsal root ganglion to deliver frataxin to the neurons and myocytes. The schematic was created with BioRender.com.

Gene editing protocol for FRDA was first optimized in FRDA patients’ lymphoblasts and healthy HSPCs using two separate gRNA surrounding the GAA expansion region and editing efficiency ranged from 39.8 to 61.9% and 32.6–49.8%, respectively (Rocca et al., 2020). Directed removal of *FXN* GAA expansion was then tested in CD34^+^ HSPCs from FRDA patients, and we demonstrated gene editing of 12.1–55.9% with a mean of 29.66%, across multiple patients, and with excision of >1,000 repeats. Increased *FXN* expression was observed in the edited cells with significant correlation between the proportion of gene editing and level of *FXN* expression. Furthermore, xenotransplantation of genome edited CD34^+^ cells into non-obese diabetic (NOD) severe combined immunodeficiency (SCID) Il2rg^−/−^ (NSG) mice demonstrated that our editing approach did not alter the *in vivo* hematopoietic repopulation and differentiation capacity. Altogether, these data represent the manufacturing feasibility of a gene editing strategy for CD34^+^ cells from FRDA patients and is schematized in Figure 2. Our previous *in vivo* study in YG8R mice demonstrated that 30% of wildtype donor cell engraftment was sufficient to fully rescue the FRDA phenotype, and we obtained up to 55.9% of *FXN* of gene editing efficiency in patients’ CD34^+^ HSPCs, suggesting that this approach could reach therapeutic threshold for FRDA.

A potential issue associated with the clinical translation of CRISPR/Cas9 edited CD34^+^ HSPC is the transient overexpression of p53 due to CRISPR/Cas9-induced DSBs and its downstream negative cell cycle regulator, CDKN1A/p21, leading to cell cycle arrest for damage response proteins to repair (Schiroli et al., 2019). This results in gene edited CD34^+^ cell proliferation delay and thus potentially decrease editing efficiency after transplantation. Despite this set back, CRISPR/Cas9 mediated gene editing has demonstrated encouraging results in current clinical trials (Frangoul et al., 2021b) and has spurred research on understanding and reducing the impact of transient p53 expression in the context of gene editing-induced DSBs. Indeed, knockdown or knockout of *p53* or inhibiting its activity with small molecules partially restores cell cycle progression, and also improve gene editing (Ihry et al., 2018; Riesenberg and Maricic, 2018; Geisinger and Stearns, 2020) but long-term inhibition of p53 can increase selection of cells tolerant towards DNA damage and risk tumorigenesis, a specific concern in cells aimed for clinical translation. This had led to exploring transient silencing of *p53* or reversible inhibition of the protein. As such, transfection of mRNA encoding dominant negative mutant of p53, GSE56, in human cord blood HSPCs improved cell proliferation after Cas9-mediated DSB induction, and increased HDR efficiency by 50% without affecting the self-renewal capacity of HSPCs *in vivo* (Ferrari et al., 2020). Thus, ensuring a delicate balance between efficiency of gene editing and controlling the DNA damage-repair mechanism, while maintaining the stem cell potency of CD34^+^ HSPC, is necessary to ensure a safe and efficacious *ex vivo* gene editing method for FRDA and other diseases.

## Conclusion

Gene therapy and genome editing have transformed the treatment options for genetic disorders. Each approach comes with their own limitations and assessing the risk:benefit ratio for therapeutic intervention is highly disease-specific. Gene editing to remove the GAA expansion in the intron 1 of *FXN* for FRDA presents critical advantages, considering the toxicity of overexpression of the protein. Because of the systemic character of the disease, gene editing of patients’ autologous HSPCs appear like an attractive option as intelligent and widespread delivery vehicles to obtain a stable, sustained and regulated expression of frataxin in all the appropriate tissues. It also brings new perspectives to regenerative medicine, showing its applicability for multi-compartment disorders involving deficient mitochondrial function and addressing the pressing and systemic unmet medical need.
